# In Vitro and In Vivo Evaluation of Bioactive Compounds from Berries for Wound Healing

**DOI:** 10.1016/j.cdnut.2024.102078

**Published:** 2024-01-14

**Authors:** Stefano Vendrame, Tolu Alaba, Natalie Marchi, Panagiotis Tsakiroglou, Dorothy Klimis-Zacas

**Affiliations:** 1 School of Food and Agriculture, University of Maine, Orono, ME, United States; 2 Graduate School of Biomedical Science and Engineering, University of Maine, Orono, ME, United States; 3 Division of Hematologic Malignancies and Bone Marrow Transplantation, Sidney Kimmel Comprehensive Cancer Center, Johns Hopkins University, Baltimore, MD, United States

**Keywords:** berry, berries, phytochemicals, polyphenols, wound healing, skin injury

## Abstract

Managing chronic wounds can be challenging and have a major impact on the quality of life, due to the significant financial and psychosocial burden on the affected individuals and their families. The need for safe, effective, and cost-efficient wound healing remedies has led to the identification of naturally occurring bioactive compounds with positive effects on tissue regeneration. Berry fruits are a promising source of such compounds and may therefore prove distinctively beneficial. Here, we present a qualitative review of the available evidence specifically investigating the effects of berry extracts on in vitro and in vivo models of wound healing. The evidence shows that a variety of berry extracts significantly promote wound healing through their antibacterial, antioxidant, and anti-inflammatory properties as well as their ability to stimulate collagen synthesis, re-epithelization, granulation, and vascularization pathways. However*,* data are still insufficient to pinpoint the differential effect that individual berries may have based on their nutrient and bioactive profile, the type and frequency of application, and the dosage required. Future research is needed in view of translating the available evidence into practice for clinical wound treatment.

## Introduction

The skin is the largest organ of the human body. It consists of 3 main layers (the epidermis, dermis, and hypodermis) that constitute a protective barrier shielding the body from external stimuli or environmental factors that are potential agents of injury [1]. A wound is a physical injury that causes damage to the skin and neighboring tissues, resulting in the disturbance of their normal anatomical structure and loss of physiological functions. Wound healing is a critical process that facilitates the recovery of such damage in injured tissues; it is imperative for survival that wounds are healed quickly and without complications [2].

Damage to the skin can be classified into 2 major categories: acute and chronic wounds [1]. Acute wounds affect ∼11 million people with 300,000 hospitalizations in the United States every year [3]. These wounds are typically from a blunt or penetrating trauma damaging the skin, such as abrasions, burns, bites, cuts, scrapes, scratches, and surgical incisions. They heal within a relatively short period and with minimal complications [2].

If a skin injury is not healed after 12 wk of the initial tissue disruption, the wound is defined as chronic. The number of patients with chronic wounds is dramatically rising in the United States due to the increasing aging population and the increased prevalence of chronic diseases such as diabetes [3]. Indeed, underlying pathologies such as diabetes and hypertension disrupt the natural healing process by prolonging the inflammatory phase, increasing susceptibility to infections, forming biofilms of resistant bacteria, and interrupting dermal and/or epidermal cell responses, resulting in a failure of the wound to heal completely. Chronic wounds include diabetic ulcers, pressure ulcers, and vascular ulcers [3].

Diabetic ulcers result from prolonged diabetic conditions in patients who typically have neuropathy, vascular impairment, and hyperglycemia. Recurrent diabetic ulcers occur in 40 % of patients within 1 y after the ulcer has healed. Lower extremity amputation is a common outcome of chronically unhealed and severely infected ulcers [4].

Pressure ulcers develop due to a prolonged pressure/shearing force applied to an area of skin and are most common in patients who are immobilized. Depending on the severity, the pressure ulcer may simultaneously affect the skin, subcutaneous fat, muscle, and fascia. Stage 3 and stage 4 pressure ulcers have a high mortality rate, particularly among older patients [5].

Vascular ulcers result from insufficient vascular function due to a narrowing of the lumen and ischemia (arterial ulcers) or to damaged deep or superficial veins (venous ulcers). Vascular ulcers can be painful and take ≤12 mo to heal, and their prevalence is rising among individuals over 65 y of age [6].

## Stages of Wound Healing

Wound healing is a complex process consisting of 4 overlapping phases: homeostasis (coagulation), inflammation, proliferation, and tissue remodeling (scar formation) [2].

### Coagulation

The first response to a wound is to quickly activate homeostatic pathways to prevent further damage, preserve the integrity of tissues around the wound area, and prevent hemorrhage. Within seconds, the clotting cascade is initiated, and platelets and coagulation factors form a transient matrix on the wound to slow down or stop the bleeding, create a barrier against invasion and infection, and serve as a reservoir for the cytokines and growth factors necessary for subsequent cellular migration [7].

### Inflammation

Vasoconstricting factors such as thromboxane, leukotrienes, prostaglandins, angiotensin, and inducible nitric oxide synthase (iNOS) are released from endothelial and epithelial cells to constrict smooth muscles in the medial layer of the arteries [8]. Later during the wound healing process, vasodilators such as nitric oxide (NO), endothelial nitric oxide synthase (eNOS), prostaglandins, and vasoactive kinins will be released to allow cellular migration to the wound area, which is necessary for tissue repair [9].

Platelets release cytokines such as transforming growth factor (TGF)-β, interleukin (IL)-6, and IL-1β that create chemotactic gradients for neutrophils to migrate from the circulation and phagocytose bacteria and dead cells [10]. Neutrophils secrete fibronectin, elastin, and more cytokines, which increase chemotactic gradients to attract monocytes [11]. Monocytes differentiate into macrophages, which engulf pathogens, cellular debris, and neutrophils. These inflammatory cells release cytokines, chemokines, and growth factors needed for granular formation, angiogenesis, and tissue regeneration [12]. Chemokines secreted by macrophages recruit lymphocytes to secrete cytokines for fibroplasia [13].

### Proliferation

Inflammation, cytokines, and growth factors activate vascularization via hypoxia-inducible factors and vascular endothelial growth factor (VEGF) pathways [14]. Immune cells within the fibrin matrix secrete growth factors such as fibroblast growth factors (FGFs), platelet-derived growth factor, and VEGF for endothelial cell migration [15]. Through the activities of eNOS, endothelial progenitor cells migrate from bone marrow and hair follicles to form vascular plexi, supplying nutrients and oxygen to the wound [16]. After about 4 d, granular expression of newly formed tissues is present in the clot. Fibroblasts migrate and proliferate inward from the dermis to the edge of the wound via epidermal growth factor activity [17]. Collagen type III, elastin, fibronectin, and proteoglycans are released by fibroblasts to form connective tissue and replace the temporary matrix with a new extracellular matrix [18]. These proteins and accumulated growth factors from macrophages also activate epithelial migration to cover the wound, forming a new stratified epidermal layer. Hair follicles and sweat glands supply stem cells that differentiate into keratinocytes [19], proliferate, migrate inward to cover the dermis, retract, and attach to the underlying basal membrane of the new epidermis. Thereafter, fibroblasts differentiate into myofibroblasts that connect with extracellular proteins like actin and myosin to contract the wound [20].

### Remodeling

Skin tissues subsequently undergo remodeling to restore functional anatomical structures within the wound and form a mature scar. During this stage, fibronectin, IL-10, and other anti-inflammatory factors are released to resolve inflammation [21]. The extracellular matrix secretes antiangiogenic factors such as matrix metalloproteinases (MMPs), interferons, endostatin, and thrombospondin-1 to regress the capillaries and form matured vascular density [22]. For complete wound healing, MMP degrades collagen type III, whereas TGF-β and FGF activate fibroblasts to secrete collagen type I and form mature scar tissue [23].

## Potential Effects of Berry Bioactive Compounds on Wound Healing

The botanical definition of berries is different from the commercial one (for example, strawberries, raspberries, and blackberries are not botanical berries, whereas grapes, tomatoes, and bananas are). For the purpose of this review, the term berry is used under its commercial meaning, commonly referring to the small, juicy fruits of red-blue-purple color and sweet-sour-tart taste, mostly belonging to the families Ericaceae and Rosaceae, and whose English name often ends in -berry. Such categorization also has the important advantage of grouping fruits characterized by distinctive nutritional features, and in particular their high phenolic acid (PA), proanthocyanidin, and anthocyanin (ACN) content [24].

Polyphenols are a large class of secondary metabolites produced by plants as a defense mechanism against environmental stress that are biologically active in the human body and exert a variety of health promoting activities [25]. In particular, 2 classes of polyphenols are found at high concentrations in berries and have been investigated for their implication in the wound healing process: PAs and flavonoids [26].

Several studies have investigated the roles of PAs in skin protection and repair of different types of wounds due to antibacterial, antioxidant, and tissue regenerative effects. For example, gallic acids have been reported to significantly regulate oxidative markers and cellular migration of keratinocytes and fibroblasts to wound area in both diabetic and nondiabetic wounds [27], whereas loading a chitosan-based film with tannic acids significantly inhibited bacterial growth while promoting complete wound healing during dermal treatment [28].

Flavonoids have been shown to exert anti-inflammatory, antiproliferative, antibacterial, antioxidant, and cytotoxic activities, which are critical for wound healing [29]. They have also been shown to act as modulators of collagen synthesis, promote wound contraction, and increase production of epithelial cells that facilitate skin regeneration as well as growth factors such as VEGF to promote wound healing [30]. Finally, flavonoids have been reported to protect against UV radiation-induced skin damage [31]. Indeed, extracts from flavonoid-rich lemonberry and elderberry have been shown to increase photoprotection against UV skin damage [32,33].

Micronutrients, such as vitamins and trace elements, can also influence the healing process of a wound. Their administration has been reported to reduce the risk of pressure wounds and to reduce the size and depth of ulcers in bedridden patients with stage 3 or 4 pressure ulcers [34]. Micronutrients that have been shown to promote wound healing include zinc, manganese, magnesium, iron, copper, and most vitamins [35].

Vitamin C is abundant in blueberries and strawberries [24]. The main involvement of vitamin C in the wound healing process lies in it being a key ingredient for collagen synthesis in the skin, but its antioxidant activity and mediation of cellular apoptosis have also been implicated [36]. Indeed, during the inflammatory phase of a wound, vitamin C facilitates neutrophil apoptosis whereas during the proliferative phase, it is essential for the synthesis, secretion, maturation, and degradation of collagen [37].

Vitamin A is especially abundant in blackberries [24]. It promotes collagenase and boosts monocytes and macrophages at the wound site, reducing inflammation. It also promotes epithelial cell differentiation while stimulating the immune system as well as promotes the growth and differentiation of skin epidermis around the wound [35].

Group B vitamins are more abundant in cranberries, raspberries, and blueberries [24]. In one study, a vitamin B complex supplement promoted rapid wound healing in type 2 diabetic rats with a significant decrease in the expression of proinflammatory genes at the wound area [38].

Vitamin E is abundant in cranberries and blackberries [24]. Its involvement in the wound healing process is mostly accounted for by its antioxidant activity against lipid peroxidation and against the formation of malondialdehyde to promote wound closure [35].

Vitamin K is of primary importance for wound healing due to its fundamental involvement in the blood-clotting process, and it is abundant in some berries, such as the star gooseberry [39].

Zinc is a cofactor for metalloenzymes required for collagen synthesis, immune function, cell proliferation, tissue repair, and wound healing [40]. To promote wound healing, zinc has been shown to act as a cofactor for lysyl oxidase enzymes involved in the synthesis of collagen. It also promotes protein formation and the development of tissue around the wound area [40]. Iron, magnesium, and copper are also essential for the wound healing process due to their involvement in the formation of collagen and by acting as cofactors for enzymes involved in wound repair [35]. Raspberries and blackberries are especially rich sources of these minerals [24].

Manganese, which is especially abundant in blueberries, has been shown to stimulate the proliferation of keratinocyte and fibroblast cell lines via the expression of integrins [41] and to exert significant antioxidant and antimicrobial activity at the wound site [42].

## Scoping Review of the Available Literature

As described above, berry fruits are characterized by an abundance of bioactive compounds with wound healing potential. To determine whether this composition has practical effects on the wound healing process, we conducted a scoping review of the scientific literature, with a focus on in vitro and in vivo studies specifically investigating the effects of berry fruits and their extracts or fractions on wound healing.

To this aim, the scientific literature was searched using the PubMed database (updated November 2023) using the following key words: (berry OR blueberr∗ OR raspberr∗ OR cranberr∗ OR strawberr∗ OR bilberr∗ OR chokeberr∗ OR blackcurrant OR açai) AND (“wound healing” OR “skin injury”) and restricting the search to studies published over the last 15 years in the English language. A total of 450 records were screened for relevance by scanning the titles and abstracts, leading to the identification of 41 records. Studies related to cancer-related or UV-related lesions were excluded. Following full text examination, 25 studies were selected for extraction of the following data: type of study, berry used, type of extract, tested concentrations, wound model used, and relevant outcomes related to wound healing.

A descriptive, qualitative review of the available evidence is presented in the following paragraphs.

## In Vitro Models of Wound Healing with Berry Extracts

The effects of several berry extracts and fractions that have been investigated in vitro by measuring the migration rates of different cells involved in the wound healing process as well as antibacterial, antioxidant, and anti-inflammatory activity is summarized in Table 1 [37,[43], [44], [45], [46], [47], [48], [49], [50], [51], [52], [53], [54]].

**TABLE 1:** Summary of in vitro studies on wound healing with berry extracts

Family	Berry	Model	Fraction(s)	Concentration	Overall effect^1^	Reference
*Arecaceae*	Açai berry (*Euterpe oleracea*)	HNF cells	Water extracts	0.1, 0.3, and 1 mg/mL	Increased fibroblast migration rate and expression of associated genes	[43]
*Ericaceae*	Bog blueberry (*Vaccinium uliginosum*), crowberry (*Empetrum nigrum*), and lingonberry (*Vaccinium vitis-idaea*)	HDF cells	Crude extracts, anthocyanin and proanthocyanidin fractions	50 μg/mL	Increased fibroblast migration rate; decrease in oxidative stress and inflammation	[44]
*Ericaceae*	Wild blueberry (*Vaccinium angustifolium*)	HUVECs	Phenolic acid extract and anthocyanin:phenolic acid combination	0.002, 60, and 120 μg/mL 8:8 and 60:60 μg/mL	Modulation of endothelial migration and associated factors	[45]
*Ericaceae*	Wild blueberry (*Vaccinium angustifolium*)	HUVECs	Phenolic acid extract and anthocyanin:phenolic acid combination	0.002, 60, and 120 μg/mL 8:8 and 60:60 μg/mL	Modulation of endothelial cell tube formation and associated factors	[46]
*Rosaceae*	Blackberries (*Rubus* spp.)	HaCat keratinocyte cells	Anthocyanin extract	30 μM	Increased keratinocyte migration	[47]
*Rosaceae*	Blackberry (*Rubus plicatus*) and raspberry (*Rubus idaeus*)	*Staphylococcus aureus* isolated from wound	Methanol extracts	N/A	Antibacterial, antioxidant, and anti-inflammatory effects	[48]
*Rosaceae*	Raspberry (*Rubus idaeus*)	HDF cells	Juice	1 % w/w	Increased fibroblast migration	[49]
*Rosaceae*	Raspberry (*Rubus idaeus*)	HaCat keratinocyte cell	Crude extract	50 μg/mL	Increased keratinocyte migration, antioxidant properties	[37]
*Rosaceae*	Himalayan raspberry (*Rubus ellipticus*)	Antioxidant assay	Acetone, methanol, and hot water extracts	100 μg/mL	Antioxidant properties	[50]
*Rosaceae*	*Rubus imperialis*	Murine fibroblast L929 cells	Methanolic extracts	1, 10, 100 and 1000 μg/mL	Increased fibroblast migration; decreased oxidative stress and inflammation	[51]
*Rosaceae*	Strawberry (*Fragaria x ananassa*) and blackberry (*Rubus fruticosus*)	HDF cells; mouse macrophage cell lines	Crude extracts, anthocyanin and proanthocyanidin fractions	1, 5, and 50 μg/mL	Increased cell migration rate; decreased inflammation and oxidative stress	[52]
*Phyllanthaceae*	Indian gooseberry (*Phyllanthus emblica*)	Human keratinocyte cells and HeLa cells	Crude extracts	60 mg/mL	Increased keratinocyte migration and antioxidant activities	[53]
*Myrtaceae*	Jaboticaba Brazilian berry (*Plinia cauliflora*)	Murine fibroblast L929 cells	Hydroalcoholic extract	0.5, 5, 25, 50, and 100 μg/mL	Increased fibroblast proliferation and decrease in oxidative stress	[54]

Abbreviations: HaCat, aneuploid immortal keratinocyte; HDF, human dermal fibroblast; HNF, human normal fibroblast (HS68, ATTC, American Type Culture Collection); HUVEC, human umbilical vein endothelial cell; N/A, Not Available.

1 Only statistically significant outcomes are reported.

Antibacterial activity is the first mechanism of defense in the wound healing process, and it has been repeatedly reported for berry extracts. Indeed, when the effect of blackberry and raspberry extracts was tested in bacterial cultures of different *Staphylococcus aureus* strains isolated from wounds, both extracts showed significant antibacterial activity after 24 h of treatment. Blackberries had a stronger effect than raspberries, and leaf extracts had stronger activity than fruit extracts, likely due to their higher phenolic content and antioxidant strength [48].

Antioxidant and anti-inflammatory effects have also been consistently reported for several berry extracts. Different extracts from raspberry leaves (with methanol, acetone, or hot water) were tested at different concentrations in radical scavenging assays. Although all extracts had significant antioxidant properties, methanol extracts (MEs) showed a stronger effect against NO and superoxide radicals, whereas water and acetone extracts were more effective against 2,2-diphenyl-1-picrylhydrazyl [50].

The effects of crude extracts (CEs), anthocyanin fraction (AFs), and proanthocyanidin fraction (PFs) from blackberry and strawberry were tested on human dermal fibroblasts (HDFs) and mouse macrophage cell lines for LPS-induced oxidative stress and inflammatory markers. Both strawberry CE and PF, but non AF, significantly decreased reactive oxygen species (ROS) after 24 h [52]. Compared to strawberries, blackberries had an even more significant effect on oxidative stress, with the PF having the strongest ROS reduction, followed by the CE and the AF*.* Regarding inflammation, both strawberries and blackberries significantly decreased cyclooxygenase (COX)-2 and iNOS gene expression, with the strongest effect induced by the PF, followed by the CE. Interestingly, raspberry PF was more effective against IL-6 whereas blackberry PF was more effective against IL-1β (43 %). Consequently, the use of strawberries and blackberries for anti-inflammatory properties may be specific to targeted pathways and require a combination of fractions, especially the PF [52].

The fibroblast migration and proliferation rate is another key parameter for effective wound healing, and several berry extracts have been shown to increase it. In the abovementioned study, for example, fibroblast migration after 48 h of treatment increased with all fractions, with the strongest effect observed for the AF of blackberries and the CE of strawberries [52].

The effect of polyphenol-rich CEs from crowberry, bog blueberry, and lingonberry, as well as specific AFs and PFs, was tested on fibroblast migration, oxidative stress, and inflammation in HDF cells. After 24 h of treatment with 50 μg/mL of each fraction, AF and PF in bog blueberry and crowberry significantly decreased ROS, whereas the CEs of all berries were less effective against oxidative stress. All bog blueberry extracts and PFs from all berries significantly reduced expression of the COX-2 gene, with the most significant anti-inflammatory effect from the bog blueberry PF. All berry extracts significantly decreased iNOS expression, with the PF being the most effective. Bog blueberry CE had the most significant increase in fibroblast count, followed by PF and CE from lingonberry and AF from bog blueberry. However, in the HDF migration assay, crowberry had the lowest fibroblast migration at 24 h [44].

The effect of Brazilian berry hydroalcoholic extract at different concentrations (0.5, 5, 25, 50, and 100 μg/mL) was tested on murine fibroblasts with hydrogen peroxide-induced oxidative stress. After 24 h of treatment, a significant effect against oxidative stress was observed at concentrations of 25 μg/mL or higher, with the highest concentration showing the strongest effect. Concentrations of 25 μg/mL or higher also promoted a significant increase in fibroblast proliferation at 48 h, whereas the 100 μg/mL concentration already induced significant fibroblast migration after 24 h [51].

Extracts of *Rubus imperialis* leaves and branches at different concentrations (1, 10, 100, and 1000 μg/mL) were tested on fibroblast migration, wound closure, oxidative stress, and inflammation in murine fibroblast L929 cells. After 24 h of treatment, the 10 μg/mL extract induced the most significant fibroblast migration, followed by the 100 μg/mL and 1 μg/mL concentrations. The 100 and 1000 μg/mL concentrations had the strongest antioxidant activity against 2,2-diphenyl-1-picrylhydrazyl. The 10 and 100 μg/mL concentrations had the most significant increase in neutrophil efferocytosis, which promoted the conversion of proinflammatory M1 cells to anti-inflammatory M2 cells. The 10 μg/mL extract also had the highest protection against mitochondrial toxicity, suggesting that this is the most effective concentration for both fibroblast recruitment and anti-inflammatory and antioxidant effects during wound healing [51].

In a wound assay, the effect of water extracts from açai berry at different concentrations (0.1, 0.3, and 1 mg/mL) has been tested in human normal fibroblast cells (HS68, ATCC, American Type Culture Collection) on cell migration rates and associated genes (procollagen-1, MMP-1, and fibronectin). The study reported that treatment with 1 mg/mL açai berry water extract had the highest rate of fibroblast migration to wound sites and the most significant increase in fibronectin mRNA expression and decrease in MMP-1 mRNA expression [43].

In a scratch wound assay on HDF cells, raspberry extract (1 % w/w) was incorporated into a hyaluronic acid base cream (enriched with 0.75 % vitamin E and 2 % green tea) and tested on fibroblast migration, showing a significant increase after both 24 and 48 h of treatment [49].

Keratinization is another an important mechanism involved in re-epithelization during wound healing. In a similar study, the effects of raspberry extract on keratinocyte migration (at a concentration of 5 μg/mL) and oxidative stress (at 50 μg/mL) were examined. Both at 24 and 48 h of treatment following the scratch wound assay, there was a significant increase in keratinocyte migration, while antioxidant activity was significantly increased only after 24 h of treatment [37].

ACN, cyanidin-3-glucoside, and malvidin-3-glucoside fractions (30 μM) from blackberry were tested on keratinocyte migration rate in HaCat keratinocyte cells. Five hours after treatment, ACN and cyanidin-3-glucoside fractions had significantly increased keratinocyte migration, and after 10 h, all blackberry fractions significantly increased migration rate. The cyanidin-3-glucoside fraction had the most rapid wound healing effect, decreasing healing time by about 50 % compared with control [47].

A significant increase in the number and size of keratinocyte colonies was also observed after 2 wk of treatment with Indian gooseberry CEs at 60 mg/mL in HeLa cells. The treatment also showed a strong protective effect against oxidative stress, likely due predominantly to the abundance of vitamin C [53].

It is important to consider that the dose and type of extracted fraction may have important differential impacts on the wound healing process mechanisms. For example, wild blueberry fractions of PAs (0.002, 60, and 120 μg/mL), ACN (at 60 μg/mL), and a combination of both (PA+ACN, at 8:8 and 60:60 μg/mL) were tested on human umbilical vein endothelial cells for endothelial migration and associated factors (gene expression and protein levels of Ras-related C3 botulinum toxin substrate 1 [RAC1] and Ras Homolog Family Member A). Endothelial cell migration rate was significantly increased by all PA treatments, with the strongest effect at the 0.002 μg/mL, followed by the 60 μg/mL and 120 μg/mL concentrations, and then by PA+ACN at 60:60 g/mL and 8:8 g/mL. Conversely, ACN treatment alone inhibited endothelial migration. Expression of RAC1 and Ras Homolog Family Member A significantly increased following all treatments, with the strongest effect for the PA treatment at 0.002 μg/mL and 60 μg/mL [45].

In a subsequent study with the same experimental design, the different fractions (except for 120 μg/mL PA) were also tested on angiogenesis and associated factors (gene expression and protein levels of eNOS, RAC(Rho family)-alpha serine/threonine-protein kinase [AKT1], and VEGF1). Endothelial tubular formation was increased by PA and PA+ACN treatments, with the strongest effect at the 0.002 μg/mL and 60 μg/mL PA concentrations. Conversely, ACN treatment decreased angiogenesis. Expression of AKT1 and eNOS was downregulated by exposure to ACN and PA+ACN, whereas VEGF and AKT1 expression was upregulated by exposure to PA, with the strongest effect at the 0.002 μg/mL concentration. Protein levels of AKT1 increased with PA at 0.002 μg/mL and PA+ACN at 60:60 μg/mL [46]. Overall, these data show that endothelial cell migration and angiogenesis are differentially modulated based on the type of phenolic components and their concentrations. The data also suggest that higher phenolic concentrations do not necessarily exert stronger effects and may actually become less beneficial.

In conclusion, the available in vitro evidence indicates that a variety of berries may exert beneficial effects on the wound healing process due to a combination of different mechanisms; in particular, their antioxidant, anti-inflammatory, and antimicrobial properties and their promotion of fibroblast migration and proliferation rate, keratinocyte migration, and vascularization. The potential effects were determined largely from the profile of bioactives in the fraction, concentrations of berry extract, type of solvent they are dissolved in, and the treatment exposure time of the cells used for the study.

## In Vivo Models of Wound Healing with Berry Extracts

The effects of several berry extracts on the wound healing process have also been tested in vivo, using incision and excision models of wounds to measure closure rate and associated parameters such as collagen deposition, vascularization, inflammation, and antibacterial properties, as summarized in Table 2 [37,43,50,51,[55], [56], [57], [58], [59], [60], [61], [62]].

**TABLE 2:** Summary of in vivo studies on wound healing with berry extracts

Family	Berry	Model	Fraction(s)	Concentration	Overall effect^1^	Reference
*Arecaceae*	Açai berry (*Euterpe oleracea*)	Sprague–Dawley rats	Aqueous extracts	1 %, 3 %, and 5 %	Increased rate of wound contraction; increased re-epithelization and vascularization; decreased inflammation	[43]
*Arecaceae*	Açai berry (*Euterpe oleracea*)	Sprague–Dawley rats	Aqueous extracts	1 %, 3 %, and 5 %	Increased wound closure and collagen deposition; fewer mast cells	[55]
*Arecaceae*	Açai berry (*Euterpe oleracea*)	CD1 mice	Whole berry	500 mg/kg BW (oral administration)	Modulation of the Wnt/β-catenin pathway, decreased inflammation	[56]
*Ericaceae*	Blueberry (*Vaccinium corymbosum*)	Sprague–Dawley Rats	Anthocyanin	2 % w/v	Increased wound contraction, collagen content, and vascularization; decreased inflammation	[57]
*Ericaceae*	Cranberry *(*Vaccinium macrocarpon*)*	Sprague–Dawley Rats	Oil extract	100 mg/kg	Increased wound contraction, granular formation, and antimicrobial and anti-inflammatory activities	[58]
*Rosaceae*	Himalayan raspberry (*Rubus ellipticus*)	Diabetic Wistar rats	Methanol extract	1 % and 2 % w/w	Increased antibacterial activity, epithelization, and collagen formation	[50]
*Rosaceae*	Raspberry *(Rubus idaeus)*	Mice	Crude extract	20, 40, and 80 mg/kg (oral administration)	Increased wound contraction	[37]
*Rosaceae*	*Rubus imperialis*	BALB/c mice	Methanolic extract	1 % and 2.5 % w/v	Increased wound contraction and collagen and fibroblasts on wound; reduced inflammation	[51]
*Rosaceae*	*Rubus sanctus*	Sprague–Dawley Rats and Swiss albino mice	Hexane, chloroform, ethyl acetate, and methanol extracts	1 % w/v	Increased wound contraction, re-epithelization, and collagen deposition	[59]
*Adoxaceae*	Dwarf elderberry (*Sambucus ebulus*)	Wistar rats	Methanol extracts	2 % and 5 % w/v	Increased angiogenesis, fibroblast count, and epithelial and granular thickness	[60]
*Phyllanthaceae*	Star gooseberry (*Sauropus androgynus*)	Diabetic Sprague–Dawley Rats	Alcoholic extract in cream base	2 % w/v	Increased rate of wound closure, collagen deposition, and vascular growth factors; decreased inflammation	[61]
*Cactaceae*	Barbados gooseberry (*Pereskia aculeata*)	C57BL/6 mice	Methanol extracts and hexane fraction	5 % w/v	Increased wound contraction, collagen content, and blood flow and decreased inflammation	[62]

Abbreviation: BW, body weight.

1 Only statistically significant outcomes are reported.

The effects of açai berry water extracts at different concentrations (1 %, 3 %, and 5 %) were tested on skin wounds in Sprague–Dawley rats, showing an increased rate of wound closure after 6 and 12 d, although controls showed no difference at day 18. The expression of genes related to re-epithelization and vascularization (VEGF, collagen-1, and fibronectin) increased, whereas the expression of genes related to inflammation (MMP-1 and IL-1β) decreased. The strongest effect of all markers was found at the highest concentration [43]. In a subsequent study with a similar experimental design to study the effect of the extracts on oral wounds, the 3 % and 5 % concentrations increased wound closure rate at days 3 and 6 of treatment. This effect was further confirmed by histological analysis, which showed a significant decrease in mast cells and increase in collagen deposition [55].

ACN extract from blueberry incorporated at 2 % w/v into a hyaluronic acid base was tested in a rat excision wound model for 4, 8, 12, and 16 d, resulting in a significant increase in wound closure rate, vascularization, and collagen content at all treatment durations. After 4 d of treatment, the most significant increase in VEGF protein was observed as well as the most significant decrease in NFκB and iNOS expression. Expression of JAK and IL-10 genes was increased compared with controls only at days 4 and 8, whereas the effect disappeared with longer treatment [57].

Treatment of Sprague–Dawley rats for 13 d with an oil extract from cranberry (100 mg/kg body weight) significantly increased wound closure, granular formation, and collagen content and decreased inflammation. It also resulted in significant inhibition of *Staphylococcus aureus*, *Escherichia coli*, and *Klebsiella pneumoniae* at the wound site compared with controls [58].

MEs from elderberry were tested at different concentrations (2 % and 5 % w/v) in Wistar rats subjected to full-thickness wounds, resulting in a significant increase in angiogenesis, fibroblast count, and epithelial and granular thickness. Interestingly, although both concentrations had beneficial outcomes, the strongest effect was observed with the lower concentration of elderberry extract, and the effect on fibroblast count and neovascularization was most significant after 7 d of treatment compared with 14 and 21 d of treatment [60].

MEs from *Rubus imperialis* leaves and branches at different concentrations (1 % and 2.5 % w/v) were tested in BALB/c mice with surgically induced skin lesions, resulting in increased would contraction rate and an increase in fibroblast and collagen deposition for tissue regeneration at all concentrations. Meanwhile, oral treatment of mice with 100 mg/kg of the ME resulted in a significant decrease in neutrophils and leukocytes in the air pouch model of localized inflammation [51].

Different extracts (prepared with hexane, chloroform, ethyl acetate, and methanol) of *Rubus sanctus* at 1 % w/v were tested in linear incision and excision wound models in Sprague–Dawley rats and Swiss albino mice. Only the ME resulted in a significant increase in wound contraction, by over half at day 6 of treatment and by over 80 % at day 12 compared with controls. ME also had the most significant re-epithelization, but the other extracts determined the strongest increase in fibroblasts count [59].

The effects of 5 % w/v ME or hexane fraction (HF) from Barbados gooseberry (*Pereskia aculeata*) was tested on skin wounds in C57BL/6 mice. Increased wound closure was found at days 5, 7, and 10 with HF treatment, and a weaker and more delayed effect was found with the ME. Likewise, only HF treatment significantly increased blood flow on day 3 and collagen count on day 14. Inflammatory markers were also decreased, but surprisingly, microscopy analysis showed a decrease in vascular formation following treatment [62].

The alcoholic extract of star gooseberry at 2 % w/w was tested in diabetic Sprague–Dawley rats subjected to skin wounds infected with methicillin-resistant *Staphylococcus aureus*. After 14 d of treatment, with 2 daily topical applications, the rate of wound closure was significantly increased as well as skin tensile strength, collagen deposition, VEGF expression, and skin thickness. A beneficial effect on inflammation was also observed, with decreased C-reactive protein concentrations and COX-2 expression [61].

Raspberry leaf MEs at different concentrations (1 % or 2 % w/w) were tested in diabetic Wistar rats and showed significant wound healing properties in incision, excision, and *Staphylococcus aureus*-induced infected wound models. Both concentrations led to complete epithelialization within 13 to 19 d. Wound closure increased by almost 70 % after 12 d of treatment with the 1 % extract and doubled with the 2 % concentration. The 2 % extract also had the strongest antimicrobial effect on the infected wound area. The extract also resulted in increased antioxidant activity as measured by glutathione, glutathione peroxidase, and catalase levels but had no effect on superoxide dismutase level [50].

Although most wound healing studies test berry extracts in topical applications in the form of creams or ointments, dietary treatment also has the potential of being beneficial. When different concentrations (20, 40, and 80 mg/kg) of crude raspberry extracts were fed to mice subjected to skin wounds, all concentrations resulted in significantly decreased wound area after 14 d of dietary treatment, with the strongest effect for the higher dose [37]. Following oral administration of açai berry (500 mg/kg) for 6 d to CD1 mice subjected to full-thickness excisional wounds, a significant modulation of the activity of the Wnt/β-catenin pathway—which is involved in the inflammatory process and in growth factor activity during wound healing—was observed. In particular, expression of Wnt3a and β-catenin cellular accumulation decreased, as well as decreased levels of TNF-α and IL-18 and NFκB activation. [56].

In conclusion, the use of berry extracts has shown promising potential in in vivo models of wound healing. A multiplicity of beneficial effects has been reported, such as increased rate of wound contraction, increased re-epithelialization, increased vascularization, increased collagen formation, decreased inflammation and oxidative stress, and antimicrobial properties at the wound site.

Although most studies testing multiple concentrations of extracts found the strongest effects with the highest concentration, some studies found better outcomes with lower dosages. Interestingly, when the effects on would healing were investigated at multiple time points during the treatment, the strongest effects were often observed during the first week of treatment, and then tended to disappear, suggesting that longer treatments may be unnecessary.

Overall, these data indicate that a more careful comparative analysis of different concentrations and treatment durations should be performed in future studies to determine the optimal combination of these 2 parameters, in view of potential clinical applications.

## Concluding Remarks

**FIGURE 1:**
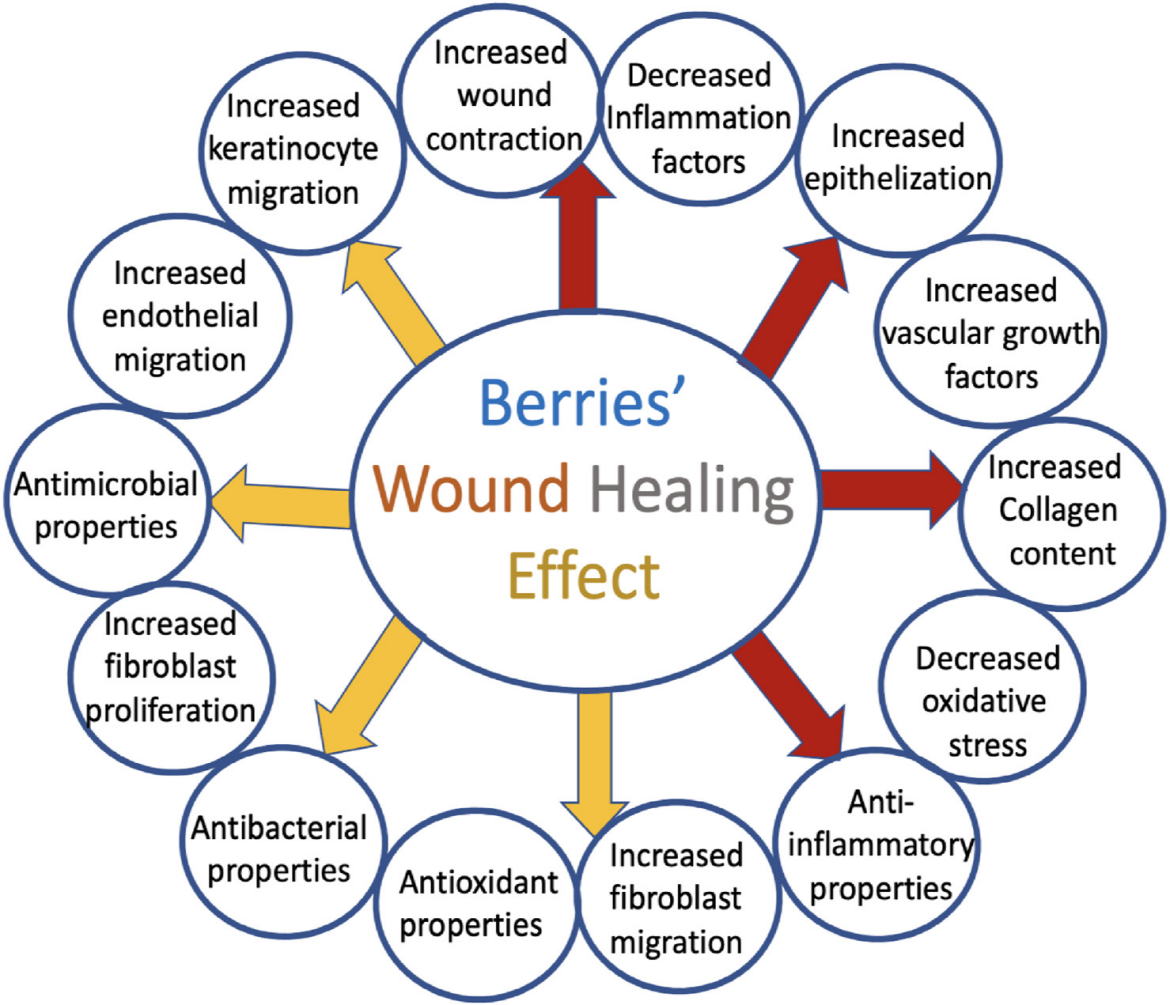
Reported in vivo and in vitro effects and pathways of berries during wound healing. Light-colored arrows indicate documented in vitro effects whereas dark-colored arrows indicate in vivo effects.

A number of studies in recent years has clearly documented that a variety of berry extracts significantly promote several mechanisms involved in the process of wound healing and repair. As summarized in Figure 1, they have been shown to exert antibacterial, antioxidant, and anti-inflammatory properties as well as the ability of promoting collagen synthesis, re-epithelization, granulation, and vascularization pathways*,* all essential for effective tissue regeneration [1]. Such therapeutic potential has been linked in particular to their distinctive content of micronutrients and phytochemicals, such as phenolic compounds, flavonoids, ACNs, and proanthocyanidins.

However, the available evidence is derived from a wide variety of different berries and the different types of extracts and fractions isolated from them. The number of available studies is still largely insufficient to determine or even speculate on the differential effect of individual berries or to tease out the contribution of their specific components in terms of nutrients and bioactives and their synergistic interactions.

Since the specific mechanisms and pathways involved may differ among berries and extracts, future research is needed to comprehensively understand the specific mechanisms and pathways involved and how this information could be translated into clinical wound treatment. In particular, attempting to pinpoint the best berries (or their combinations), parts (fruits, leaves, etc.), extracts (which fractions and type of solvent used for extraction), vehicle (gel, cream, powder, nanocarrier, etc.), type of administration (topical treatment or oral administration), concentrations, and treatment duration is of paramount importance for designing appropriate clinical trials. This review provides insight into potential berry candidates for clinical trials to determine if their therapeutic potential can indeed be translated into practical applications in the clinical setting.

In conclusion, this review provided an overview of the effects of different berry extracts on wound healing pathways in both in vitro and in vivo wound models. We document a promising potential of berry extracts to promote the rate of wound closure and skin regeneration at different stages during the wound healing process. We conclude that the available evidence provides a sufficient basis for the development of clinical trials to pinpoint how this information could be used for the development of safe, effective, and cost-efficient wound healing treatments.

## Author contributions

The authors’ responsibilities were as follows – DK-Z: conceptualized and designed the review; TA, NM, PT: conducted the literature search and drafted the first version of the manuscript; SV: completed the literature search and drafted the second version of the manuscript; DK-Z: revised the manuscript; and all authors: read and approved the final manuscript.

### Conflict of interest

The authors report no conflicts of interest.

## Funding

The authors reported no funding received for this study.
